# Barriers to Social Participation among Lonely Older Adults: The Influence of Social Fears and Identity

**DOI:** 10.1371/journal.pone.0116664

**Published:** 2015-02-23

**Authors:** Johanna C. Goll, Georgina Charlesworth, Katrina Scior, Joshua Stott

**Affiliations:** Research Department of Clinical, Educational and Health Psychology, University College London (UCL), London, United Kingdom; Medical University Vienna, AUSTRIA

## Abstract

**Introduction:**

Loneliness among older adults is a major public health problem that may be associated with processes of social participation and identity. This study therefore sought to examine the relationship between social participation and identity in a sample of lonely older adults living independently in London, England.

**Method:**

An inductive qualitative approach, based on semi-structured interviews and thematic analysis, was employed.

**Results:**

Participants commonly spoke of barriers to social participation that have been reported elsewhere, including illness/disability, loss of contact with friends/relatives, lack of a supportive community, and lack of acceptable social opportunities. However, novel findings were also derived. In particular, participants commonly minimised the difficulties they faced alone, and described attempts to avoid social opportunities. These behaviours were linked to fears about engaging in social participation opportunities, including fears of social rejection and/or exploitation, and fears of losing valued aspects of identity.

**Discussion:**

It is concluded that social participation amongst lonely older people will not improve through the removal of previously reported barriers alone; instead, older peoples’ beliefs, fears and identities must be addressed. Suggestions for implementing these findings within community organisations are provided.

## Introduction

Loneliness among older adults is recognised as a major public health problem [1]. Evidence points to associations between late-life loneliness and reductions in social participation [2]. Whilst mechanisms leading to reduced social participation are poorly described, literature indicates the potential influence of identity processes [3,4]. This study examined the relationship between social participation and identity in a sample of lonely older adults living independently in London. In what follows, each of the main concepts addressed (loneliness, social participation, identity) are outlined, before the study aims are presented.

### Loneliness

Loneliness describes the distress that accompanies a perceived lack of social relationships [2,5]. Older adults experience increased levels of loneliness [6–7], with prevalence estimates ranging from 10 to 50% [2,5,8]. A growing literature indicates that lonely older people experience increased morbidity and mortality [9–12]. Therefore, late-life loneliness is increasingly recognised as a major public health problem [1,13–14].

A small literature has delineated risk factors for late-life loneliness, including: poor health/disability, death of a partner, living alone, lower activity levels, loss of social contacts and lack of appropriate transport [5,15]. Additionally, longitudinal studies that have sought to examine the development of loneliness highlight the importance of *relative* rather than absolute losses in health and social resources over time [6,16–17]. However, much further research is required in order to understand and address late-life loneliness.

### Social Participation

Given this limited knowledge, it is helpful to examine other processes that contribute to late-life loneliness, such as reduced *social participation*. Social participation is commonly regarded as involvement in interpersonal interactions outside the home, including social/leisure/community activities, and work [18–19]. Whilst limited reductions in late-life social participation may carry certain benefits [20], compelling evidence suggests that significant reductions lead to poor health outcomes [21,22]. Evidence suggests links between social participation and loneliness in later life. For example, like loneliness, reduced social participation is common in older adult populations [23,24] and is associated with similar negative health outcomes [19,21,22,25]. Additionally, interventions to increase social participation may lead to reductions in loneliness [26]. Finally, given longitudinal findings discussed above [16], loneliness may be associated with difficulties maintaining previous levels of social participation following relative losses in health and/or social resources. Given this literature, it might be useful to explore the uptake of social opportunities among lonely older people.

Previous research has delineated risk factors for reduced late-life social participation, including: higher age, illness/disability, lower socioeconomic status, and ethnic minority status [23,27,28]. Additionally, qualitative studies have begun to describe older adults’ subjective experiences of barriers to social participation, including: perceived danger in the neighbourhood, ageism, lack of finances, lack of confidence, lack of opportunities that support preferred identities, and difficulties adapting to ageing [29–31]. However, these preliminary findings require development in further research.

### Social Participation and Social Identity

A separate literature suggests that reduced late-life social participation may be mediated by *social identity*, i.e., the self-conceptualisations that individuals derive from their group memberships (e.g., woman, teacher, hill-walker, Christian) [32,33]. There is evidence for strong links between social identity and utilisation of healthcare [3]; for example, healthcare is more readily accepted when a receiver shares a preferred social identity with a provider, and a lack of shared social identity may lead to service refusal [34,35]. This suggests that an older man may not wish to attend a group populated by mainly women because this would contradict his male identity. Alternatively, an older person who identifies as a “care-provider” may not want to attend a support group for fear that he/she would become a “care-recipient”. Moreover, widespread ageism [36,38] may lead individuals to avoid groups for older people in case they become identified as “old” and thus stigmatised [4].

### Study aims

This study examined the relationship between social participation and social identity among lonely older adults living independently in London. A qualitative interview-based approach was chosen given the limited knowledge of this topic to date. The research questions were:

1. What are the barriers that prevent lonely older adults from accessing opportunities for social participation?
2. How do lonely older adults respond to these barriers?
3. How, if at all, are these barriers and responses related to their social identities?

## Methods

### Ethical Approval

This study was carried out with the approval of University College London Research Ethics Committee, according to guidelines established by the Declaration of Helsinki [39]. Details of the approved procedure are given below.

### Quality Assurance

This interview-based study adhered to accepted criteria for reporting qualitative research (COREQ) [40]. Of particular note, the following strategies were employed: (i) to guard against bias, a reflexive stance was adopted in which the research team regularly assessed the impact of their own biases and the possibility of alternative interpretations; (ii) the stages of the research process and the perspectives of the researchers were presented as transparently as possible, in order that readers might be able to judge the value and transferability of findings; (iii) a team-based approach was employed so that no single perspective held undue influence; and (iv) portions of the analytical process were performed independently by two researchers (JG, KS) and subsequently checked for convergence.

### Recruitment

Recruitment took place via four voluntary sector organisations situated in urban and multicultural boroughs of inner-city London (within the M25 motorway boundary): three separate Age UK organizations; and an independent charity that provides a befriending service for older adults (which remains anonymous for reasons of confidentiality). The approved recruitment procedure was as follows. Staff at each of the charitable organisations were asked to identify individuals already known to them who met the following inclusion criteria: 60 years or older; judged by staff to be currently accessing none/few social opportunities; judged by staff to be experiencing loneliness and/or social isolation; able to communicate effectively in spoken English (fluency not required). Staff introduced the study to identified individuals using a recruitment leaflet (see S1 File). Where individuals gave their permission to be contacted by the research team, the first author (JG) telephoned individuals to give more details and to check that they were eligible and willing to participate, and able to give informed consent [41]. Where these conditions were fulfilled, JG arranged to visit the individual’s home on a subsequent day. At the beginning of research visits, JG read the study information sheet (see S2, in S1 File) with participants, and allowed as much time as required for questions. JG then rechecked that participants were still willing to take part and verified that they could give informed consent, before asking them to complete a written consent form (see S3, in S1 File). Recruitment ceased when the research team considered that a rich data set had been acquired, and that additional interviews were adding little novel information.

### Data collection

All participants took part in a semi-structured interview conducted by JG, which lasted between 60 and 90 minutes and was audio-recorded. The full interview schedule (S4) is provided in S1 File. The interview aimed to explore (i) participants’ social identities, (ii) participants’ experiences of, responses to, and wishes for social opportunities, and (iii) any links between these factors. After the interviews, participants were invited to verbally provide a range of demographic information (age, ethnicity, details of any current illnesses and/or disabilities, occupational history; see interview schedule (S4) in S1 File) and to complete three quantitative measures to assess (i) loneliness [42], (ii) social interaction [43], and (iii) depression [44]. Finally, participants’ socioeconomic status (SES) was estimated using: (i) Standard Occupational Classification (SOC) [45]; and (ii) Neighbourhood deprivation statistics (NDS) [46]. For the SOC, where a participant reported a spouse with a higher-rated occupation, this was recorded since it was assumed to be a better indicator of the couple’s overall SES. For the NDS, data were taken from the *Office for National Statistics* website (http://www.neighbourhood.statistics.gov.uk/dissemination/), and derived in the following way: each participant’s postcode was entered, the option of *Lower Layer Super Output Area* was selected, and the statistic labelled as *All People of Working Age Claiming a Key Benefit* was recorded (found under the section labelled *Key Figures for Economic Deprivation*). Participants were compensated for their time with a £10 gift voucher.

### Analysis

The analysis was performed using Thematic Analysis (TA) [47], situated within the epistemological framework of Constructivist Grounded Theory (CGT) [48]. CGT principles guided the analysis towards an interpretation of participants’ personally constructed belief systems, via a focus upon their words *and* actions. Additionally, CGT principles encouraged the authors to adopt a reflexive stance, so that they could consider the impact of their own meanings upon findings. Of note, all four authors are clinical psychologists who aim to tackle barriers to wellbeing in later life, and particularly ageism; thus, CGT helped the authors to keep this positioning in mind, and to look for alternative perspectives throughout.

Analysis of transcribed interviews was facilitated by use of the software package *Dedoose* [49]. Firstly, JG coded all data items that held relevance to the research questions. Subsequently, another author, KS, independently coded two transcripts; this procedure validated the codes identified by JG and additionally introduced some novel codes. Next, these two authors together collated codes into themes, and themes into clusters. Finally, all four authors met to assess and refine the themes and clusters in relation to the raw data, making minor adjustments where appropriate.

## Results

### Participants

Twenty-nine individuals (10 males, 19 females) were referred to the study. Of the 10 referred males, five declined because they were not interested in taking part. Of the 19 females, two could not be contacted and seven did not meet inclusion criteria. The final sample of ten females and five males ranged in age from 62 to 100 years (mean = 79, SD = 12); further sample characteristics are provided in Table 1. All participants lived alone, with the exception of one female who lived with her husband who had severe dementia. Whilst levels of social interaction varied [43], 12 of the 15 participants reported relatively restricted social networks, and all but two reported no engagement with social groups (P12 and P15 went to church weekly; [43], questions 7 & 8). All were classified as lonely [42]. Additionally, all reported some form of illness or disability. The sample was ethnically and socioeconomically diverse.

**Table 1:** Participant characteristics.

Participant number	Age group	Gender	Network Typology (PANT)	Loneliness	Depression	Ethnicity	Disability	Illness	Socioeconomic Status	
									SOC	Neighbourhood deprivation (%)
P1	late	M	PR	Severe	-	White British	Mobility, vision	-	1-Management	5
P2	late	F	PR	Moderate	-	White British	-	Memory, history of falls	3-Technical^1^	15
P3	late	F	PR	Severe	Mild	White British	Mobility, vision	Bowel condition	8-Operative	14
P4	early	F	LSC	Moderate	Mild	Central Asian	Mobility	-	2-Professional	16
P5	late	M	PR	Severe	Moderate	White British	Mobility	-	4-Administrative	31
P6	mid	F	WCF	Very Severe	Moderate	White British	-	Chronic depression	2-Professional^1^	7
P7	early	F	PR	Moderate	Moderate	Black Caribbean	Mobility, vision	Diabetes	2-Professional	18
P8	late	F	FD	Severe	Mild	White British	Mobility	-	1-Management^1^	13
P9	late	F	PR	Moderate	Moderate	White British	Mobility, registered blind	-	2-Professional	13
P10	early	F	PR	Very Severe	Severe	White British	Mobility	History of stroke	9-Elementary	22
P11	late	F	PR	Moderate	Mild	White British	Mobility	History of falls	4-Administrative	14
P12	mid	M	LI	Severe	Severe	South-East European	Mobility	History of cancer	5-Skilled trades	20
P13	early	M	PR	Moderate	-	White British	Cerebral Palsy	-	8-Operative	19
P14	late	M	PR	Moderate	Severe	White British	Mobility	History of stroke	9-Elementary	33
P15	mid	F	WCF	Severe	-	Black Caribbean	Vision	Diabetes	5-Skilled trades	23

***KEY***:*-*, *absence of depression*, *disability or illness*;

^*1*^, *occupational classification based on spouse’s occupation, see methods; **Age group**; “early” 60–69, “mid” 70–79 years, “late” 80+ years; **Depression**, see methods; **F**, female; **Network Typology (PANT)**, see methods; **Loneliness**, see methods; **M**, male; **Memory**, subjective memory impairment; **Mobility**, age-related mobility difficulties; **Neighbourhood deprivation**, index of neighbourhood economic deprivation, see methods; **SOC**, Standard Occupational Classification, see methods; **Vision**, age-related visual impairment*.

### Themes

The analysis led to the generation of 14 themes, which were grouped into four clusters (see Table 2). An example of a coded excerpt (S5), and the constituent codes for each theme (S6), are presented in S1 File. In what follows, themes are described and illustrated with quotes. Participants are identified by codes corresponding to Table 1, and *Int*. denotes the interviewer. For ease of reading, repeated words and non-words have been deleted from quotes, superfluous segments have been replaced with an ellipsis (…), and connecting words have been inserted (in square brackets []).

**Table 2:** Clusters and themes.

Clusters	Themes
**1. Overt barriers**	1.1 Illness and disability
	1.2 Loss of friends and family
	1.3 Loss of community
	1.4 Perceived lack of social opportunities
**2. Responses to barriers**	2.1 Minimising the difficulties of loneliness
	2.2 Not seeking social interaction
	2.3 Avoiding social opportunities
	2.4 Relying on the telephone
	2.5 Keeping busy with solitary activities
**3. Social fears**	3.1 Fear of rejection
	3.2 Fear of exploitation
**4. Fear of losing preferred identities**	4.1 Fear of losing “independent” identity
	4.2 Fear of losing “youthful” identity
	4.3 Fear of losing preferred social identity

### Cluster 1: Overt barriers

Themes in this cluster described overt barriers to social participation, including illness/disability, loss of friends and family, loss of a local community, and a perceived lack of social opportunities. Data indicated that each of these barriers consisted of an interplay between objective components (e.g. actual lack of social opportunities) and perceived components (e.g. perception of lack of social opportunities).

**1.1 Illness and disability.** Almost all participants said that their illnesses and disabilities led to a range of practical issues that made social participation challenging, including low energy levels, difficulties utilising transport, difficulties managing symptoms, and problems mobilising.

P4 I have weakness in my legs [and] I get tired extremely soon, so from that point of view [it’s] sort of difficult in trying to go out.

In addition to these practical issues, participants explained that *anxieties* about their health/disability issues discouraged them from social participation; they worried about falling, being unable to cope with symptoms whilst not at home, and the unpredictability of accessible transport.

P3 If you go out by [accessible taxi] … you wonder if they’re gonna turn up.

P11 I think I’m gonna fall over at [any] moment.

**1.2 Loss of friends and family.** Around two thirds of participants related their low interaction levels to the absence of old friends and neighbours who had died, and the absence of family members who had moved away.

P14 The majority of blokes I knew went to the pubs, well they’re dead and buried.

P12 [My family] phone me up sometimes but they can’t come here, they are very far.

This lack of existing social contacts was compounded by a reluctance to form new relationships with “strangers”, who participants felt would not understand them or offer genuine support.

**1.3 Loss of community.** Around half of the participants mourned the loss of an “old community” in which residents had supported one another. They felt abandoned by “uncaring” neighbours and therefore disinclined to pursue local social opportunities.

P5 If you was missing … [neighbours] would knock on the door and just find out if you was alright. You don’t get none of that today.

P10 I don’t think I’d want to go [to a local group] … For four years I’ve been sitting here and you haven’t helped … You’re not nice people. I don’t want to know.

They associated the loss of community with a high turnover of local residents (especially the influx of younger people and non-English speakers), a perceived increase in crime, and the loss of valued social groups. Notably, perceptions of a lost community were particularly prevalent among participants from lower socioeconomic backgrounds.

P15 This [neighbour] is Polish. That one is Turkish. Who the hell do you associate with? … I’m the only original resident on this block. Everybody has changed.

P10 [Neighbourhood meetings have] finished now, because not near enough people could be bothered … There were a lot of us, but now nobody cares.

**1.4 Perceived lack of social opportunities.** Almost all participants had little knowledge of local social opportunities. The men in the sample were often unaware that social opportunities might exist, whilst the women tended to have a basic awareness of services but limited knowledge of specific opportunities. Additionally, most participants asserted that they would not like particular activities offered at groups (which they thought might typically include bingo, “light entertainment”, and chatting), or the food provided.

P3 Well I don’t mind mixing with people but, um, I don’t know what groups there are.

P14 Oh look, [social groups are] a load of crap and people all “yap yap yap yap” talking all the time.

P2 If [lunch is] put before me … [it might] just put me off. Will I hate it, their sort of food? I don’t know.

### Cluster 2: Responses to barriers to social participation

This cluster describes participants’ responses to the barriers to social engagement that they encountered. Notably, most minimised their difficulties, did not seek to increase their interaction levels, avoided social opportunities, and/or gave up on socialising altogether. Instead, they coped by relying on telephone communication and solitary activities.

**2.1 Minimising the difficulties of loneliness.** Around half of the participants asserted that they enjoyed spending time alone, and these claims might have been presented as a theme in their own right. However, without exception, all such statements were associated with contradictory ideas that indicated a need for a more complex and nuanced theme. For example, all participants who claimed to value solitude also described strong desires for more interaction. Additionally, they often inadvertently revealed the uncomfortable challenges brought by loneliness through apparent attempts to demonstrate that they could cope alone.

P9 I just take [being alone] in my stride.

P4 [Being alone] doesn’t bother me anymore.

Taken together, it appeared that participants were unsatisfied with their isolated lives, but tried to cope through emphasising the positives of solitude, and either omitting or minimising associated difficulties within conversations.

P11 Don’t feel sorry for me or anything. I mean, I’m OK … It took me a long time to get used to this but I’m getting used to it now … I think, well, you’ve seen [the world], you have to be thankful for that … So I try to make myself feel better about it all.

**2.2 Not seeking social interaction.** All participants exhibited a general lack of interaction-seeking behaviour in response to their loneliness. They tended to state that they would not consider contacting anyone (individuals or services) if they were feeling lonely. They also seemed to accept aversively low levels of social contact, without asking for anything more.

Int. Do you ever think about contacting anybody at all if you’re feeling lonely?
P5 Well there ain’t anybody much I know.
Int. You wouldn’t think about contacting any of the people that you’ve told me about?
P5 Nah.

P11 [My friend] who takes me shopping had been away, so I hadn’t been out for quite some time.

Moreover, several participants indicated that they had ceased looking for suitable opportunities to socialise, given their circumstances. They communicated a sense of hopelessness and defeat, which in some cases appeared indicative of low mood more generally. At the same time however, there was no clear association between depression and isolation and/or loneliness across the sample as a whole (Table 1).

P11 Well I just can’t be bothered … I just don’t want to make the effort. I mean, I don’t get up very early in the mornings … When I feel lonely I don’t want to do anything … [I’ve] just lost interest.

**2.3 Avoiding social opportunities.** Almost all interviews revealed strategies that participants used for avoiding social interaction opportunities. For example, participants stated that they would refuse any invitations to local groups without hesitation. They also described “putting off” interactions; some directly admitted this, whilst others gave incoherent reasons for missing social opportunities that were suggestive of delaying behaviours.

P8 I made up my mind that I was going to go to this centre, but [my son] was here. I had to cook for him, look after him, so really I couldn’t get to where I wanted to go. So that’s the reason I didn’t go, it was all to do with him …
Int. And when he went, did you go?
P8 No … then I thought … five weeks, it’s passed, and no I didn’t.

Additionally, almost all participants’ avoided social opportunities on the basis of negative predictions, e.g. that activities would not be enjoyable, or that others would not be welcoming.

Int. What do you think the [group] atmosphere would be like?
P9 Well, I can only imagine what it would be like but I don’t know from experience.
Int. No, what do you imagine it would be like?
P9 Well, I just wouldn’t feel comfortable.

**2.4 Relying on the telephone.** Around two thirds of participants stated that talking to friends and relatives on the telephone helped them to feel less lonely. For these individuals, the telephone seemed to provide a social “lifeline” that kept them going.

Int. If you’re feeling lonely do you ever think about getting in touch with anybody?
P15 Well I’m always on the phone.

**2.5 Keeping busy with solitary activities.** All participants reported using solitary home-based activities to mitigate loneliness, particularly television-watching, radio-listening, reading/writing, and doing household chores. Three participants described solitary activities as direct replacements for face-to-face interaction, and one of them claimed that she was so busy that she rarely felt lonely (P3).

P5 If I’m watching football I don’t mind … I get carried away with that, so I’m alright.
P15 I will watch the TV in the evenings only for just to have a bit of noise in the house and a bit of companionship.

### Cluster 3: Social fears

Themes in this cluster suggested that participants avoided social opportunities for fear that they would be rejected and/or exploited by their peers.

**3.1 Fear of rejection.** Around half of the participants, and particularly women, feared various forms of rejection by social groups, neighbours and services, including: being excluded from group discussions/activities (particularly by pre-existing members who might be reluctant to admit newcomers), being refused help by services, and being humiliated following transgression of perceived social standards (e.g. making “mistakes” during discussions). Those who seemed to fear rejection the most reported a longstanding (and perhaps lifelong) preoccupation with this issue.

P11 I’ve met those sort of clubs before, where people stick together and they don’t want anyone new in … I don’t want to go.

P2 I don’t like being an outsider … I don’t want them not to like me … I don’t want to be scorned.

P7 Ask for help and you’re turned down. That hurts … It’s not worth the aggravation … I don’t want to ask anybody for anything, nothing … I don’t want another knock-back.

**3.2 Fear of exploitation.** Four female participants avoided social groups because they feared that members would exploit their kindness and generosity. In particular, they worried that group members whose moral standards did not match their own would “pluck their eyes out” (P15), and that vulnerable members might become burdensome.

P6 Obviously people have to tell me their problems … and I will get worried about them … I want to go to groups that will make me feel better, not burdened with more problems.

### Cluster 4: Fear of losing preferred identities

Themes in this cluster suggested that most participants avoided social opportunities due to fears that attendance would threaten valued or preferred aspects of their identities. In particular, they feared losing their “independent” and “youthful” identities, and their preferred social identities.

**4.1 Fear of losing “independent” identity.** Almost all participants emphasised independence (being capable of supporting themselves) as a valued and honourable aspect of their identities. At the same time, they equated help-seeking with dependency, incapability, and additionally amorality, because in their eyes it involved exploiting the kindness of others. Importantly, participants saw accessing community services as a form of help-seeking that would threaten their independent identities. For example, one man who struggled to look after himself avoided a lunch group for fear of losing his self-sufficiency (P12). However, six participants expressed a desire to support others, and two said that they would happily receive support if they could simultaneously support the other person. Therefore, engagement in reciprocal helping roles appeared acceptable, whilst dependency was not.

P7 I’m not asking for help. I’ll [go to a group] if I feel I can bring something.

**4.2 Fear of losing “youthful” identity.** Around two thirds of participants characterised “old” people in very negative terms, describing them as sick, disabled, dependent, incapable and decrepit. Thus, it was unsurprising that participants made frequent attempts to distance themselves from “old” people, often describing themselves as youthful and “young at heart”.

P8 I don’t really act like an old person … I’m very young at heart … When I get dressed up, I don’t think I look like some of them … But I feel sorry for old people.

Furthermore, participants imagined that groups for older people consisted of rooms of “lifeless” individuals doing nothing and waiting to die. Thus, they avoided such groups, believing that they would have nothing in common with members, and fearing that attendance might make them “old” too.

P12 I see all [these men] sleeping like that, sleeping. They all have their mouth open … No, I don’t wanna be like that. I don’t wanna go and sit in [that group] … No.

P7 If you go in a group and they’re all older … you become like them too.

Instead, participants expressed preferences to associate with youthful people who might help them to feel “young again”, but struggled to find such opportunities.

Notably, an opposite process was observed in interviews with three male participants, all aged 80 years or above: they explained that they had withdrawn from social opportunities because they viewed themselves as “too old” (P1, P5, P14).

**4.3 Fear of losing preferred social identity.** Almost all participants wished to avoid social opportunities that might contradict their preferred social identities (see Table 3). For example: a woman who described herself as socially refined avoided mixing with anyone she perceived as “common”; another woman who described herself as educated avoided a group with members she perceived as “not bright”; a man whose identity as a sports fan involved taking part in repartee eschewed mixing with people who did not offer “jolly” conversation; a Christian woman who described herself as selfless had withdrawn from church activities because she viewed other worshippers as “greedy”; a man who described himself as a “normal guy with a disability” (cerebral palsy) evaded groups in case others treated him as “different”. Instead, participants desired, but struggled to find, social opportunities that reinforced their preferred social identities. For example: participants who regarded themselves as educated desired opportunities for intellectual discussion with similarly educated others; a woman who saw herself as a caregiver wished to interact by continuing her charity work; an ex Church minister wanted to engage in church-based activities; a retired doctor wanted to socialise with other doctors.

**Table 3:** Participant social identities.

Social identities
Political/community activist, educated person
Wife, educated person, friend
Mother, friend, hobbyist
Doctor, caregiver, educated person
Husband, father, sports fan, joker
Wife, mother, educated person, socially refined person, charity founder, caregiver, friend
Professional caregiver, educated person, mother, friend
Wife, mother, educated person, socially refined person, friend
Christian, church minister, caregiver
Caregiver
Educated person, friend
Respectable family man (honourable, dedicated to care of family), father
"Normal bloke" (enjoys sport & pubs) with a disability
"Normal bloke" (enjoys sport & pubs)
Christian, church volunteer, mother, caregiver

P4 As a professional I would rather be in [a] group of professionals, because you learn a lot from them, even by talking afterwards … otherwise … you’re sort of restricted in the character and attitude of some of people.

I What would [your ideal group] look like?
P9 … A local church fellowship group …
I And the other people, what would they be like?
P9 Well, I would imagine … they would be of similar persuasion as myself.

Notably, these processes seemed to operate regardless of social identity (Table 3), ethnicity or socioeconomic status. Overall, data suggest that social participation may reduce when interaction opportunities seem to contradict preferred identities, as P15 surmised:

P15 I’m not gonna be naïve enough to think that if I join any group … that everybody’s gonna be like me. I just have to learn to fit in, and if I can’t then I don’t go.

## Discussion

This study sought to elucidate subjective barriers to social participation in a sample of lonely older adults. The most salient barriers aligned with previous research to include illness/disability, loss of contact with friends/relatives, lack of a supportive community, and lack of acceptable social opportunities [23,27,30,31]. Somewhat counter-intuitively, but in convergence with a recent study [50], participants responded to these barriers not by seeking new and accessible social opportunities, but by psychologically minimising the challenges of loneliness, avoiding social opportunities, and attempting to cope alone. However, careful exploration of the data revealed two psychological processes that may explain participants’ responses: fear of social rejection and/or exploitation, and fear of losing preferred identities. These particular barriers have not been previously described, and therefore represent a novel contribution to the literature. Here, these barriers will be discussed, and strategies for intervening will be suggested. However, the success of such interventions will depend upon careful research to evaluate their efficacy and build an evidence base upon which services may draw. An accessible summary of findings is also available (see S7, in S1 File).

### Addressing social fears

Current evidence suggested that lonely older adults avoid social participation opportunities for fear of rejection and/or exploitation by their peers. Social fears have been previously linked to loneliness and social isolation, in working-age adults [51], and lonely older people [52]. Evidence suggests that loneliness reflects a lack of perceived safety in social situations, which leads to cognitive and behavioural patterns that maintain loneliness, such as selective attention to negative social stimuli [53–54]. In view of this literature, current findings indicate that similar processes may contribute to reduced late-life social participation. If valid, this claim suggests that talking therapies that target maladaptive behaviours and cognitions, like Cognitive Behavioural Therapy (CBT) [55], might enhance late-life social participation. However, since lonely and socially fearful older people are unlikely to engage in therapy without significant support, a more effective strategy might comprise the incorporation of CBT principles into pre-existing community groups. For example, organisations might challenge fears about attendance through: emphasising the friendliness of groups, implementing a “buddy” system for new members, normalising social fears, and facilitating gradual steps towards participation. Organisations might implement such recommendations in consultation with a mental health professional such as a clinical psychologist.

### Addressing identity processes

The present findings suggest that lonely older adults avoid social opportunities for fear of invalidating their preferred identities. Firstly, in line with previous evidence, findings indicated that participants sought to uphold independent and youthful identities [56–58]. They frequently emphasised their self-sufficiency, distinguished themselves from “old” people whom they described as dependent and decrepit, and avoided opportunities for support in case this marked them as old and dependent. Secondly, participants emphasised their preferred *social identities* (self-conceptualisations derived from group memberships, e.g. caregiver, Christian, educated person, sports fan, “bloke”). In agreement with other studies [3,4], they avoided social situations that might contradict their preferred identities, and wished for (but could not find) opportunities that might instead provide identity-reinforcement. Such results must be considered within the broader context of an ageist society [36–38]. Societal discourses commonly associate youthfulness with valued traits such as independence, economic productivity and usefulness, and ageing with characteristics deemed intensely negative, like dependency and uselessness [37,59]. Thus, participants’ efforts to maintain youthfulness and independence can be viewed as attempts to preserve valued identities in a context that excludes on the basis of age alone. Additionally, attempts to maintain pre-existing social identities, which often involved being productive in some way, might also form part of this process.

These findings indicate that community groups might engage more lonely older adults by actively supporting their preferred and socially valued identities [60]. In particular, organisations might seek to reinforce “independent” identities by encouraging older people to take ownership of social opportunities. Indeed, participant ownership is likely to facilitate the development of opportunities that reflect preferred identities (for an example, see the *Men’s Sheds* movement, [61,62]). “Independent” identities might also be supported through the provision of educational and volunteering opportunities that allow older adults to develop productive, socially valued roles [63]. Additionally, organisations might develop social opportunities that de-emphasise age and therefore do not contradict “youthful” identities, perhaps through schemes that help older and younger people to connect (see *Trans-age Action*, [64]), and by facilitating all-age events. However, these strategies are unlikely to work for everyone: older people with the least resources (health, social, financial) are least likely to be able to participate [65–66]. Furthermore, the promotion of independence, productivity and youthfulness as normative ideals risks inadvertently reinforcing ageist views and devaluing older people who cannot embody these characteristics [65]. Therefore, organisations might consider promoting alternative identities that focus other areas, such as spirituality, emotional growth, artistic creativity, and relationships [65]. Indeed, the promotion of meaningful relationships might lead older adults to build valued identities based on interdependency rather than independence, so that they experience support-seeking as reinforcing rather than invalidating [67,68]. Notably, an endorsement of reciprocity among current participants indicates that older people would be receptive to such an approach. Perhaps most importantly, by relieving people of the impossible expectation of staying forever youthful and providing alternative means to construct a positive identity, community groups might increase their engagement with older people whilst simultaneously challenging ageism.

### Limitations

Current results may have been limited by difficulties in the recruitment of lonely older people [69]: the loneliest individuals may not have been referred, or may have been less likely to agree to participate. This latter problem was particularly true among the referred men, 50% of whom said that they were not interested in taking part (compared to 0% of referred females). Nevertheless, all participants were classified as lonely, over half surpassed the criterion for “severe loneliness” [42], and a satisfactory gender mix was obtained (5 males, 10 females). Thus, whilst findings were based on a significantly lonely sample, future work should investigate barriers to research participation in this population. Additionally, it is surprising that present results did not indicate any particular influence of ethnicity or culture, given the multi-cultural context. This may be due to the under-representation of people from non-White British backgrounds: whilst these people account for 55% of London’s population [70], they only constituted 25% of the current sample. This might indicate that older people from ethnic minority backgrounds were less likely to be known to the recruiting organisations. Additionally, such observations might indicate reduced social participation and increased loneliness among this population more generally [71–73]. However, these associations are likely to vary between particular ethnic groups [8] and may be mitigated by high levels of within-group participation [72]. Thus, cross-cultural differences in barriers to social participation are probable, and future research is required to explore this issue.

### Conclusions

The study illuminated subjective barriers to social participation among lonely older adults, including both commonly cited and novel factors. The novel factors suggest that reductions in late-life social participation may reflect commonplace fears of social rejection/exploitation, and fears of losing preferred aspects of identity. Taken together, present results suggest that in order to enhance social participation among lonely older people it is necessary to address individuals’ beliefs, fears, values and identities.

## Supporting Information

S1 File Combined file of supporting information. (PDF)
