# Correction: Barriers to Social Participation among Lonely Older Adults: The Influence of Social Fears and Identity

**DOI:** 10.1371/journal.pone.0201510

**Published:** 2018-07-25

**Authors:** Johanna C. Goll, Georgina Charlesworth, Katrina Scior, Joshua Stott

## Notice of republication

An incorrect version of S1 File was published in error. This article was republished on July 18, 2018 to correct for this error. Please download this article again to view the correct version.
