# Supplementary material for: Barriers to Social Participation among Lonely Older Adults: The Influence of Social Fears and Identity
Source: PLoS One. 2015 Feb 23;10(2):e0116664. doi: 10.1371/journal.pone.0116664 (PMC4338142; doi:10.1371/journal.pone.0116664)
Supplement: S1 File — (PDF) [file pone.0116664.s001.pdf]

S1. Recruitment leaflet

Name (please use capital letters):

Please tick (✓)

☐ **YES,** I would like to receive more information about taking part

☐ **NO,** I do **not** want to receive more information about taking part

How would you like to be contacted?

☐ **Phone**

Phone number: ------------------

☐ **Email** @

Email address: ------------------

☐ **Post**

Address: ------------------------

[redacted]

UCL

Can you help?

We want to hear the views of older people in London

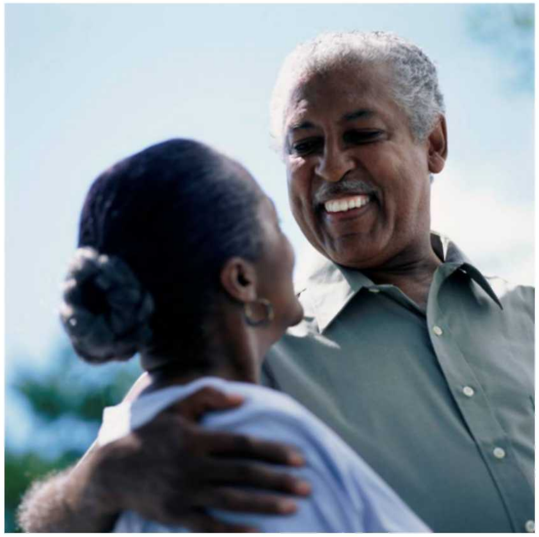

You will get a £10 supermarket voucher for your time*

S1. Recruitment leaflet (continued)

Dear London resident,

My name is Johanna.

I work at University College London (UCL).

I am trying to find out what older people think of local groups and activities.

[redacted]

I would like to talk to people who:

1. don’t go to groups and activities much

and 2. sometimes feel separate from others

I would like to find out how older people describe themselves, and how this affects their views of groups and activities.

Taking part involves talking to me about yourself and your views. We can meet in your home or a public place. It would last between 1 and 1 ½ hours.

* If you are suitable and you take part, you will get a

£10 supermarket voucher

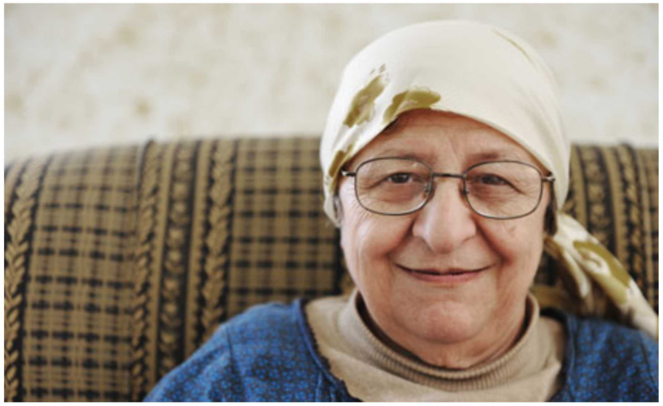

Please let me know if you are interested

- fill in and return the form (next page)
- or tell whoever gave you this leaflet
- or contact me directly (details below)

Thank you for reading this leaflet.

[redacted]

Older people and community services in London

We would like to invite you to take part in a research project.

What is the research about?

- We want to find out what older people think about groups and services in their local area. By groups and services we mean things like: social groups, lunch clubs, activity groups, and befriending services.
- We want to speak to older people who
  (1) tend **not** to go to groups and services
  and (2) sometimes **feel separate from other people**.
- We want to find out how older people describe themselves, and how this affects their choices about attending groups and services.
- We hope that this project will help us develop the kinds of services that older people in London would like.

What will the research involve?

- The main part of the research consists of **one interview** with a researcher.
- You will be asked questions about how you describe yourself as a person, and about how this affects your views of groups and services.
- The interview will last between 1 hour and 1 ½ hours, but it can be split into two shorter interviews if you prefer.
- The interview can take place in a location of your choice, such as your home, a local community building, a local café or the library.
- You will also be asked to complete **3 very short questionnaires**.
- You may also be invited to talk to us about your interview a few weeks after it has happened, to give some feedback; however, this part is **optional**.
- You will receive a **£10 supermarket voucher** to thank you for your time, if you are suitable for the project and you take part.

Room 436, Research Department of Clinical, Educational and Health Psychology
FREEPOST, University College London, London, WC1E 6BT
Tel: +44 (0)20 7679 1897 Fax: +44 (0)20 7916 1989
www.ucl.ac.uk/cehp

S2. Participant information sheet (continued)

Who can take part?

- We are looking for people who fit the following description:
    1. Aged 65 or older
    2. Able to speak English well enough to take part in an interview
    3. Tend **not** to go to groups and services
    4. Sometimes feel separate from other people
- Unfortunately, if you don't fit all 4 descriptions, you may not be able to take part.
- If you are unsure about whether you can take part, please contact the main researcher, Johanna Goll to find out more (her contact details are provided below). She will be very happy to talk to you and answer any questions.

Do I have to take part?

- You **do not** have to take part.
- It is up to you to decide whether you would like to take part. You may like to spend some time thinking about it first, or you may like to talk it over with someone else.
- Choosing not to take part will not disadvantage you in any way. For example, it will **not** affect the services that you receive.
- If you agree to take part, you are **free to stop at any time** without giving a reason.

Are there any risks involved in taking part?

- It is not expected that taking part will cause you any harm.
- The project involves talking with a researcher (to do an interview), and does not involve any other procedures, or treatments. Some people may find that the interview causes them to think or talk about personal or upsetting topics. However, you do not have to answer all the questions, and you can choose to stop the interview at any point. Additionally, all your information will be kept confidential (for more details about this, see the section below called "What will happen to the information that I give?").

Are there any benefits to taking part?

- If you are suitable for the project, and you take part, you will receive a **£10 supermarket voucher** to thank you for your time.
- Other than this, there are no direct benefits of taking part. However, it is hoped that the project will help to develop services that older people would like.
- Some people benefit from the chance to talk to someone else during an interview.
-

Room 436, Research Department of Clinical, Educational and Health Psychology
FREEPOST, University College London, London, WC1E 6BT
Tel: +44 (0)20 7679 1897 Fax: +44 (0)20 7916 1989
www.ucl.ac.uk/cehp

- Additionally, some people enjoy finding out about the results of the project when it is finished, and we will provide this opportunity for anyone who is interested.

What will happen to the information that I give?

- With your permission, your interview will be audio recorded so that it can be transcribed (written down) afterwards. Any information that would allow others to recognise you (e.g. your name) will **not** be included in the transcription. Once the transcription is complete, the recording will be deleted.
- All information (audio recordings, transcriptions, questionnaires) will be treated as **confidential**, and kept in accordance with the Data Protection Act (1998). This means that the information that you give us will be **well protected**. For example, your information will be marked with a code rather than your name, so that you cannot be recognised (information will be kept **anonymously**). Additionally, your information will be stored securely, so that only the researchers working on the project can access it.
- However, if we became **extremely** concerned about your safety or the safety of another person, we would have to break confidentiality. In this type of situation, it is our duty to get in touch with other professionals (like your GP) so that they could protect you (or another person) from harm. We would always try to discuss a situation like this with you before contacting any other professionals.

Will the results be written up and published?

The results of the project will be written up in a report and shared with local organisations including Age UK. Results may also be published in a professional journal. However, reports will include **general results only**. They will **not** contain any personal information that would allow you to be recognised. It is hoped that these reports will help organisations to develop the kinds of services that older people would like.

Who is conducting this project?

This project is being conducted by a small team of researchers from University College London (UCL), with the support of Age UK. All members of the research team have undergone **satisfactory criminal record checks** (enhanced Criminal Records Bureau (CRB) checks). Additionally, the project has been **approved by the UCL Research Ethics Committee** (Project ID Number 4454/001).

Room 436, Research Department of Clinical, Educational and Health Psychology
FREEPOST, University College London, London, WC1E 6BT
Tel: +44 (0)20 7679 1897 Fax: +44 (0)20 7916 1989
www.ucl.ac.uk/cehp

S2. Participant information sheet (continued)

- If you choose to take part, the person who you will meet and talk to is **Johanna Goll**. Her contact details are as follows.
  Post: Room 436
  Research Department of Clinical, Educational and Health Psychology
  FREEPOST University College London
  London WC1E 6BT
  Phone [redacted]
  Email:

- The person in charge of the project is **Dr Katrina Scior**. Please feel free to contact her if you have any concerns or complaints. Her contact details are as follows.
  Post: Room 436
  Research Department of Clinical, Educational and Health Psychology
  FREEPOST University College London
  London WC1E 6BT
  Email: [redacted]

Please feel free to contact Johanna (by phone, email or post) if would like any further information about the project. She will be very happy to talk to you and answer any questions.

Thank you for taking the time to read this information sheet.

Your help makes our research possible.

S3: Participant consent form

Older people and community services in London

Do you want to take part in this project? (Please tick the appropriate box)

☐ **Yes**, I would like to participate in this project.

☐ **No**, I do not want to participate in this project.

If you have answered Yes, please tick the following statements if you agree with them:

☐ I have read the Information Sheet about the project, OR it has been read to me.

☐ I understand what is involved in taking part in the project.

☐ I understand that any information I give will be kept confidential and well protected.

☐ I understand that I do not have to take part in the project if I do not want to.

☐ I understand that I may stop taking part in the project at any time without giving a reason.

☐ I have had the opportunity to ask any questions I wish.

☐ I have had enough time to think about the project.

☐ I have the names and contact details of the people running the project. I understand that I can contact them if I have any further questions or concerns.

☐ I understand that I will receive a £10 supermarket voucher after I have participated, to thank me for my time.

Name: ______________________________ Date: ________________________

Signature: ___________________________

Thank you!

Room 436, UCL Research Department of Clinical, Educational and Health Psychology
University College London, Gower Street, WC1E 6BT
Tel: +44 (0)20 7679 1897 Fax: +44 (0)20 7916 1989
www.ucl.ac.uk/cehp

S4. Interview schedule

a) Introduction

- Thanks for agreeing to take part.
- Interview focus recap. I will ask you about (1) how you describe yourself, and (2) your views and experiences of local groups and services.
- Timing recap. The interview will last 1 - 1 ½ hours. Please feel free to ask for breaks and/or multiple shorter interviews if required.
- Confidentiality recap. Your information will be unrecognisable, well-protected, and anonymous.
- Recording recap. I will use audio recording to enable transcribing. All recognisable info will be removed. After transcription, your recording will be deleted.
- Any questions?

b) Identity

Aim: to gain a sense of participant's valued social identities, and associated personal meanings.

Main Question 1: How would you describe yourself?

Sub-questions (use flexibly to help fulfil the stated aim of main question)

- What kind of person are you?
- How would you describe yourself to a person who didn't know you?
- How would other people describe you?
- What groups have you belonged to?
- What roles have you played in life?
- What are your most important characteristics?
- What does being a [valued identity] say about you as a person?
- What does it mean to you that you are/were [valued identity]?
- If I didn't know you, but I knew you are/were [valued identity], what would that tell me about you?

c) Social participation

Aim: to gain a sense of participants' beliefs and behaviours in relation to social participation opportunities (particularly groups), and how these are influenced by their valued identities (as ascertained in part B), as well as other factors.

Main Question 2: If someone invited you to join a group (or other service), what would you do?

Sub-questions (use flexibly to help fulfil the stated aim of main question)

- What would you think? What would go through your mind?
- Why would you do (or not do) that?
- What gets in the way of you going to a group/accepting a service?
- What do you think it would be like if you went to a group/accepted a service?
- What's the worst thing that might happen if you went to a group/accepted a service?
- What would it say about you as person if you went to a group/accepted a service?
- Is this related to being a [valued identity from part B]?

Main Question 3: If you could wave a magic wand, and create your perfect group (or other service), what would it be like?

Sub-questions (use flexibly to help fulfil the stated aim of main question)

- What kind of people would be there?
- What would you do?
- What makes this particularly appealing for you?
- Is this related to being a [valued identity from part B]?

S4. Interview schedule (continued)

d) Loneliness
Aim: to gain a subjective sense of how the participant typically responds to loneliness.

Main Question 4: Lots of people feel lonely from time to time. If you felt lonely, what would you do?

Sub-questions (use flexibly to help fulfil the stated aim of main question)

- Would you contact anyone?
- Would you think about contacting any local services or groups?
- Why would you do (or not do) that?
- What gets in the way of contacting [person/service]?
- What would it say about you as person if you contacted [person/service]?
- Is this related to being a [valued identity from part B]?

e) Demographic information
What is your age?
How would you describe your ethnicity?
Do you suffer from any illnesses?
Do you have any disabilities?
What is/was your occupation? Can you describe your role?

f) Quantitative Measures
Measures (see below) are administered with support from the interviewer as required.

g) Debriefing

- How did you find the interview?
- Do you have any questions about any of the things we talked about?
- Was there anything we said that has left you feeling concerned or unsettled in any way? Would you like more information about anything that we talked about?
- Do you have any questions about the research project or what will happen to your information?

S5. An excerpt from a coded transcript

	Transcript	Codes
I	If somebody said to you, Mr X, would you like to come to this club, what would you do or say?	
P	Nah	Refusing social invitations very quickly
I	You'd say no	
P	I wouldn't wanna, I wouldn't wanna know, no	
I	Can you tell me a bit about that, why would you say no?	
P	Cos I don't think they would be the same as what like I said they [my friends] used to be	Not wanting to mix with people who are not like me
I	Right	
P	I don't think they would be the same as like we used to be	
I	What do you think they would be like?	
P	Well it depends on whether, what it was all about wouldn't it really, what whatever whatever community they was, or associated with, you know what did they do, or what do they do for the people	
I	Mm, have you ever been invited to a group, or has anybody ever suggested for you to join a group?	
P	Well yeah I had a, somebody from the council been, wanted me to go to a a to a centre, but I told em what I just told you, I worked too in em, I didn't wanna go	Deciding not to interact based on negative predictions<br>Avoiding identification with old/sick/disabled people<br>Not wanting to mix with people who are not like me
I	You worked in them?	
P	Well taking the handicapped people	
I	And what is it about them that you didn't wanna go?	
P	Well they were, hard to say, it's hard to say that, they were handicapped unfortunately, but they didn't sort of mix or talk the way you used to do it, therefore, to me, there was no point in going	
I	Ah, how did they mix and talk?	
P	Pardon	Avoiding identification with old/sick/disabled people<br>Predicting that people at groups will be "lifeless"<br>Not wanting to mix with people who are not like me<br>Deciding not to interact based on negative predictions<br>Not wanting to mix in a way that is not like me
I	What did you see happen there? What was it like when you saw it?	
P	Well they just sat there and just looked at one another and er I wanted to be more jolly than that, there was no er no enjoyment in going to that at all, make you as miserable as sin, I know I'm old myself, but I can still mix with people, even now	
I	Yeah, yeah	
P	I mean if my friends were able to come here and say we're taking you out, I'd go	
I	OK	Wanting to mix with people I already know
P	And enjoy myself cos I know I would enjoy meself, so as I say, if me friends or anybody were to come here and say come on we're gonna take you out I would enjoy it yeah	
I	And which friends would it be that you would like to go with	
P	Pardon	
I	Which friends are they?	Wanting to mix with people I already know<br>Wanting to mix with people who are like me
P	They're friends from the [football team], er all the friends I mix with is [football team], that I've associated with over the years, going to many good times with them, and if they were to come here, and out I would go go go	
I	And what do you think you would do with them?	
P	Pardon	Wanting to mix with people I already know<br>Wanting to mix with people who are like me<br>Wanting to mix in a way that is like me
I	If they came here and you went with them, what do you think you would do together?	
P	Well, wherever they took me out, whether for a meal or or something and we'd have a laugh and a joke, and all that, you can't do otherwise can you, you go for a meal or what have you, and then you have a laugh, probably talk about old old times what we used to do	

N.B. The social identity of the participant in this extract was described as: husband, father, sports fan, joker.

KEY: I, interviewer; P, participant.

S6. Themes and constituent codes

Cluster 1. Overt barriers	
**Illness and disability**	Worrying about ability to manage mobility/health issues when out<br>Feeling that health/mobility issues make it difficult to go out<br>Feeling that health/mobility issues make it impossible to go out<br>Feeling that going out is too much effort<br>Feeling unable to go out due to caregiving role<br>Being so preoccupied with health/mobility problems that I can't think about social interactions<br>Difficulty managing transport
**Loss of friends and family**	Having less interaction with family than desired<br>Losing interaction with a deceased spouse<br>Losing interaction opportunities because friends/neighbours have died, become ill/disabled or moved away<br>Having less support from others than desired<br>Losing spouse as a "bridge" to social interaction<br>Wanting to mix with people I already know<br>Perceiving that there's no-one to contact/help<br>Wanting (and lacking) someone else to come out with me<br>Needing (and lacking) a friend to give me a lift<br>Being reluctant to seek support from formal services<br>Not wanting to interact with strangers/professionals/services
**Loss of community**	Perceiving a loss of community<br>Perceiving a downfall of local services<br>Experiencing less interaction with neighbours than desired<br>Feeling unsafe in the local neighbourhood<br>Perceiving a downfall of society<br>Associating neighbourhood problems with the presence of other ethnic groups
**Perceived lack of social opportunities**	Being unaware of available groups/services<br>Predicting that I won't like/be able to do offered activities<br>Predicting that I won't like/be able to eat offered food

S6. Themes and constituent codes (continued)

**Cluster 2. Responses to barriers**	
**Minimising the difficulties of loneliness**	Minimising/hiding my lack of interaction<br>Preferring own company/not wanting to interact<br>Accepting current situation
**Not seeking social interaction**	Accepting inadequate social interaction without asking for any more<br>Not asking for support<br>Feeling hopeless/defeated with regards to socialising<br>Perceiving going out/socialising as impossible
**Avoiding social opportunities**	Putting off interaction<br>Refusing social invitations very quickly<br>Deciding not to interact based on negative predictions
**Relying on the telephone**	Interacting by means other than face-to-face
**Keeping busy with solitary activities**	Engaging in solitary activities
**Cluster 3. Social fears**	
**Fear of rejection**	Fearing being let down<br>Fearing that others at the group might gossip/talk about me<br>Withdrawing from social situations due to social fears<br>Fearing social "failure"/being shamed/humiliated<br>Fearing exclusion/rejection<br>Fearing having to talk about personal matters
**Fear of exploitation**	Fearing that interaction will harm rather than help me

S6. Themes and constituent codes (continued)

Cluster 4. Fear of losing preferred identities	
**Fear of losing "independent" identity**	Feeling undeserving of support<br>Not wanting to be a burden<br>Wanting to remain self-sufficient<br>Wanting to support others
**Fear of losing "youthful" identity**	Feeling that I am "too old"<br>Avoiding identification with old/sick/disabled people<br>Predicting that people at groups will be "lifeless"<br>Avoiding being around "old" people because it will make me "old"<br>Seeking proximity to “young” people because it will make me “young”<br>Fear/experience of discrimination/stigmatisation/rejection on basis of age/disability/illness
**Fear of losing preferred social identity**	Wanting to mix with people who are like me<br>Perceiving a lack of people/services that are for people like me<br>Wanting to mix in a way that is like me<br>Not wanting to mix in a way that is not like me<br>Perceiving a conflict between society and people like me<br>Not wanting to mix with people who are not like me<br>Wanting my old life/sense of self back<br>Being unable to continue with activities that are like me<br>Engaging in solitary activities that are like me

LONDON'S GLOBAL UNIVERSITY

UCL

Barriers to social participation among lonely older adults

Loneliness is common among older people, and is widely recognised as a major public health problem because it leads to poorer health and earlier deaths. Lonely older people tend to show lower levels of participation in social and community events outside the home than expected, and this may contribute to their loneliness. Therefore, this research sought to investigate why lonely older people do not often engage with available interaction opportunities.

Key findings

- The barriers that prevent lonely older people from accessing social opportunities are not only practical, but also psychological.
- Lonely older people report that they avoid social opportunities, and do not attempt to increase their interaction levels. Additionally, some admit to giving up on socialising altogether.
- Lonely older people avoid social opportunities in part because they fear social rejection, humiliation and exploitation by their peers.
- Lonely older people also avoid social opportunities because they fear losing the self-conceptualisations or *identities* that they value. In particular, they fear losing their "independent" and "youthful" identities, as well as the identities that they have developed in occupational or social arenas throughout their lives (e.g. "educated person", "sports fan", "bloke", "caregiver").
- In this report, suggestions are provided for strategies that community organisations might employ to tackle these issues and increase social participation among lonely older people.

Background

Loneliness describes the distress experienced when an individual has fewer (or poorer quality) social interactions than desired. Loneliness is common among older people, and is widely recognised as a major public health problem because it leads to poorer health and earlier deaths. Lonely older people tend to show lower levels of participation in social and community events outside the home than expected, and this may contribute to their loneliness. Therefore, this research sought to investigate the barriers that prevent lonely older people from attending social and community opportunities. It particularly aimed to explore lonely older people's beliefs, motivations, and preferred ways of describing themselves (preferred identities), in order to understand their responses to interaction opportunities.

Findings are based on in-depth interviews that were conducted with 15 lonely older people living independently in inner London during 2013/14. Interviewees came from a wide range of social, cultural and economic backgrounds. Qualitative data analysis techniques (Thematic Analysis) were used to interpret findings.

The research was conducted by a team from The Research Department of Clinical, Educational and Health Psychology, University College London (UCL). The team were: Dr Johanna Goll, Dr Joshua Stott, Dr Katrina Scior, and Dr Georgina Charlesworth. The research was conducted in collaboration with community partners: Age UK Haringey, Age UK Bexley, Age UK Waltham Forest, and The Dorcas Befriending Project. For more details, please contact: j.goll@ucl.ac.uk.

Barriers to social participation among lonely older adults

Findings

Barriers to social and community participation, as described by lonely older people, were grouped into four themes:

1. **Overt barriers**
   The first theme comprised overt barriers that have been frequently reported elsewhere. These barriers included: illness and disability, lack of appropriate transport, loss of friends and family, loss of a cohesive local community, and a perceived lack of social opportunities.

2. **Responses to social opportunities**
   The second theme indicated that interviewees tried to hide the difficulties of being lonely, did not seek to increase their interaction levels, avoided social opportunities, and/or gave up on socialising altogether. Instead, they coped by relying on telephone communication and solitary activities in the home.

3. **Social fears**
   The third theme indicated that interviewees avoided social opportunities for fear that they would be rejected, shamed, humiliated and/or exploited by their peers.

4. **Fear of losing preferred identities**
   The last theme described how interviewees avoided social opportunities due to fears that attendance might challenge the ways in which they liked to describe themselves, or in other words, their preferred identities.
   i. Firstly, they valued independence, and feared that social opportunities might lead them to lose their "independent" identities, making them "incapable" and "dependent" instead.
   ii. Secondly, they characterised "old" people in very negative terms (e.g. sick, disabled, dependent, incapable, decrepit) and feared that attendance at groups for older people would threaten their "youthful" identities, and make them "old" too.
   iii. Thirdly, they worried that available social opportunities might conflict with the valued identities they had developed in social and occupational contexts throughout their lives (e.g. "educated person", "sports fan", "bloke", "caregiver"). For example, women who described themselves as "socially refined" or "educated" wanted to avoid mixing with anyone who seemed "common" or "not bright". Similarly, a man whose identity as a "sports fan" involved lots of joking and banter did not want to mix with people who he described as "lifeless".

Interview Quotes

(R - Researcher, I - Interviewee)

R. Do you ever think about contacting anybody at all if you're feeling lonely?
I. Well there ain't anybody much I know.
R. You wouldn't think about contacting any of the people that you've told me about?
I. Nah.

I. Don't feel sorry for me or anything. I mean, I'm OK … It took me a long time to get used to [loneliness], but I'm getting used to it now.

R. What do you think the [group] atmosphere would be like?
I. Well, I can only imagine what it would be like but I don't know from experience.
R. No, what do you imagine it would be like?
I. Well, I just wouldn't feel comfortable.

I. I've met those sort of clubs before, where people stick together and they don't want anyone new in … I don't want to go.

I. I don't like being an outsider … I don't want them not to like me … I don't want to be scorned.

I. Ask for help and you're turned down. That hurts … It's not worth the aggravation … I don't want to ask anybody for anything, nothing … I don't want another knock-back.

I. I don't really act like an old person … I'm very young at heart … When I get dressed up, I don't think I look like some of them … But I feel sorry for old people.

I. I see all [the group members] sleeping like that, sleeping. They all have their mouth open … No, I don't wanna be like that.

I. I'm not gonna be naïve enough to think that if I join any group … that everybody's gonna be like me. I just have to learn to fit in, and if I can't then I don't go.

Barriers to social participation among lonely older adults

Conclusions & Recommendations

Conclusions 1. Findings suggest that social participation amongst lonely older people may *not* be improved by tackling commonly recognised barriers alone (e.g. problems of disability, transport, loss of family/friends). Instead, it appears necessary to address psychological processes including beliefs, fears, values and identities.

Conclusions 2. Lonely older people appear to show maladaptive patterns of beliefs and behaviours that prevent them from accessing social opportunities. For example, interviewees disclosed strongly held beliefs that participation might lead to rejection, and reported the use of avoidance behaviours to evade or delay participation.

Recommendations 1. *It is suggested that community organisations might engage lonely older people by actively addressing their maladaptive beliefs and behaviours. One approach that might be employed here is that of Cognitive Behavioural Therapy (CBT). For example, following CBT principles, organisations might: emphasise the friendliness and inclusiveness of groups, attempt to normalise social fears, implement "buddy" systems so that potential members meet pre-existing members, help individuals take gradual steps towards participation, and encourage people to "test out" groups with no obligation to join.*

Conclusions 3. Lonely older adults appear to avoid social opportunities for fear of compromising the self-conceptualisations or identities that they value. In particular, they fear losing their "independent" and "youthful" identities, as well as the identities that they have developed in occupational or social arenas throughout their lives (e.g. "educated person", "sports fan", "bloke", "caregiver").

Conclusions 4. These findings must be considered within the broader context of our ageist society, in which *youthfulness* is commonly associated with independence, economic productivity and usefulness, whilst *ageing* is commonly associated with dependency and uselessness. Thus, lonely older people's efforts to maintain youthfulness and independence can be viewed as attempts to preserve culturally valued identities.

Recommendations 2. *Organisations might address these issues as follows:*

1. *They might develop social opportunities that reinforce older people's preferred identities and therefore encourage participation (e.g. the Men's sheds movement)*
2. *They might develop social opportunities that de-emphasise age and thus avoid contradicting "youthful" identities (e.g. all age events)*
3. *They might create opportunities that depend on older people's active contributions and thus reinforce their "independent" identities, perhaps by supporting activities that are designed and run by older people themselves, or by providing educational and volunteering opportunities*
4. *To avoid excluding clients for whom youthfulness and independence are not possible, they might seek to promote alternative positive identities involving, for example, spirituality, artistic creativity, emotional growth, relationships, and inter-dependency between friends*
5. *They might continue work to tackle ageism in society more broadly, so that older people may access services without fear of prejudice or discrimination*

Recommendations 3. *Organisations might develop and implement these recommendations in consultation with a mental health professional such as a clinical psychologist. Additionally, given the lack of knowledge in this field, it is of paramount importance that organisations conduct research to evaluate outcomes and build evidence upon which practitioners may draw.*
